# Influence of thermal-cycling or staining medium on the surface properties and color stability of conventional, milled, and 3D-printed base materials

**DOI:** 10.1038/s41598-024-80380-8

**Published:** 2024-11-22

**Authors:** Ruo-Jin Zhang, Lan Zhao, Lu-Xiang Yu, Fa-Bing Tan

**Affiliations:** 1 College of Stomatology, Chongqing Medical University, Chongqing, 400015 China; 2 Chongqing Key Laboratory of Oral Diseases and Biomedical Sciences, Chongqing, 401147 China; 3 Chongqing Municipal Key Laboratory of Oral Biomedical Engineering of Higher Education, No.7, Shangqingsi Road, Yuzhong District, Chongqing, 401147 China

**Keywords:** Thermal cycling, Staining media, Base resin materials, Surface properties, Color stability, Biomaterials, Dental diseases, Dentistry

## Abstract

The study of denture base resin fabricated by digital technology with surface properties or color stability remains limited. In this study, thermal cycling and staining media (distilled water, artificial saliva, green tea, and Coca-Cola) immersion were used to simulate the intraoral environment to assess the surface properties and color stability of CAD/CAM (milled) and 3D-printed base resin materials, the conventionally polymerized base served as the control group. After thermal cycling, all groups showed increased surface roughness, contact angle (i.e. hydrophilicity) and color difference (∆E), the 3D-printed group had the most significant increase among the 3 groups (*P<*0.001). While there were no significant difference (or the difference is very small) between the conventional and milled groups. After 7 and 30 days of immersion in four staining media, the ∆E values remained highest in the 3D-printed group (∆E ≥ 3.34) (*P<*0.001), exceeding the clinically acceptable threshold (∆E = 2.7) at 30 days. Additionally, all groups showed significantly higher ∆E values after 30 days compared to 7 days (*P<*0.05). The 3D-printed group exhibited a rougher surface, poorer hydrophilicity, and reduced color stability compared to the conventional or milled groups, indicating that further improvements are needed before clinical application.

**Supplementary Information:**

The online version contains supplementary material available at 10.1038/s41598-024-80380-8.

## Introduction

Resin denture bases are essential components of removable partial dentures (RPDs) and removable complete dentures (RCDs). For decades, the fabrication of denture base resins has relied on conventional lost wax-flasking-compression molding methods, which have evolved alongside advances in the properties of polymethyl methacrylate (PMMA) resins and processing technologies^1,2^. However, the fundamental principle of molding and polymerizing PMMA resins under pressure has remained unchanged. Recently, with the advent of computer-aided design/computer-aided manufacturing (CAD/CAM) technology, including milling and 3D printing, the materials and methods used for denture base resins have undergone significant changes. Subtractive techniques create the denture base resin by milling pre-polymerized PMMA discs. However, milling is costly in terms of material and time, and presents challenges in processing complex substrate structures. The latest additive manufacturing, or 3D printing, involves the continuous layering of liquid resin material on a support structure, followed by curing using visible light, UV light, heat, or laser to achieve CAD-specified denture morphology^3^. As a result, 3D-printed dentures are believed to be more efficient, consistent in quality, reduce the number of patient visits, and enhance the patient experience^4,5^.

The intraoral environment is thermodynamically dynamic due to temperature changes caused by ingested food and beverages^6^. Oral temperatures have been reported to fluctuate between 20 and 50 times daily, averaging 10,000 fluctuations per year^7^. Dentures are frequently exposed to these temperature fluctuations, which may cause surface degradation of the denture base^8–10^. Therefore, it is crucial to evaluate the performance of denture base materials in conditions that simulate the intraoral environment.

Denture surfaces must be highly polished before placement in the patient’s mouth. A rough and uneven surface, combined with the adhesion of food particles and other debris, can cause patient discomfort. A study^11^ found that rough denture surfaces serve as prime sites for the accumulation of plaque, pigments, food residues, and necrotic oral tissues, potentially leading to prosthodontic stomatitis. Taşın et al.^12^ compared the effects of different aging media on the surface roughness of denture base samples, revealing that conventional bases exhibited the highest surface roughness, while the 3D-printed group had the lowest values among the tested materials. However, another study^13^ that employed manual scrubbing and thermal cycling processes found no significant differences in changes in roughness and surface topography between 3D-printed and milled samples. These findings highlight a gap in research data needed to guide the practical application of 3D-printed denture base resins.

A previous study^14^ has demonstrated that surface roughness also influences the hydrophobicity of denture base surfaces. Contact angle measurement is commonly used to determine the hydrophobicity of these surfaces, providing a qualitative description of the degree of wetting that occurs when a non-volatile liquid (e.g., water) comes into contact with a solid surface. A contact angle of zero indicates complete wetting (hydrophilic surfaces), while a contact angle greater than zero signifies partial wetting, where the liquid spreads over a limited area and maintains a droplet shape on the surface (more hydrophobic surfaces)^15^. Steinmass et al.^16^ found that most CAD/CAM dentures have smoother surfaces, lower contact angles, and are more hydrophilic than conventional dentures. Conversely, a study by Al-Dwairi et al.^17^ reported that conventional thermal polymerization produced lower contact angles and higher hydrophilicity compared to the CAD/CAM group. Another study^18^ comparing the surface and mechanical properties of conventional and 3D-printed denture base resins revealed that the conventional thermo-polymerization group had lower mean contact angles than the 3D-printed group, indicating better hydrophilicity. However, these studies did not include a comparison of the impact of artificial thermal cycling on the hydrophilicity of conventional thermo-polymerization, CAD/CAM (milled), and 3D-printed denture base resins.

Color stability is another crucial property of denture base materials. Ideally, the color of the denture base should blend seamlessly and remain stable with the color and appearance of the gingiva and mucosal soft tissues. However, common beverages such as tea, coffee, red wine, and colored substances in food can cause discoloration of the denture in the presence of temperature changes in the mouth. Clinically, discoloration of the denture base can affect aesthetics, cause sensory discomfort, and even lead to psychological distress for the patient. Although many studies^19–24^ have evaluated the color stability of different denture base resin materials using conventional thermal-polymerized bases as a control, some^19–21^ did not compare milled dentures or 3D-printed resins, and others^22–24^ did not consider environmental factors in different staining media. While some studies^19,20^ examined the color stability of different denture base resins in various staining media, their methodologies did not simulate oral temperature changes to establish artificial thermal cycling conditions. The inconsistent results from following studies can cause confusion in clinical practice. Alfouzan et al.^20^ assessed the color difference of 3D-printed bases after thermal cycling and immersion in different staining media (coffee, lemon juice, Coca-Cola, and artificial saliva) and found that the 3D-printed bases had less color difference, indicating better color stability compared to conventional bases. However, Gruber et al.^19^ used a similar experimental approach to evaluate the color stability of milled, 3D-printed, and conventional heat-polymerized PMMA resins after thermal cycling and immersion in staining medium, reaching the opposite conclusion that the 3D-printed resin group exhibited the greatest color difference.

In summary, existing research has not resolved the debate over the surface properties and color stability of conventional, milled, and 3D-printed denture base resins under thermal cycling conditions. There is also a lack of detailed research on the surface properties and color stability of 3D-printed resins in more complex intraoral environments, considering factors such as the number of cycles, staining medium, and immersion time. Currently, artificial thermal cycling (in water baths at different temperatures) to simulate intraoral temperature changes (30 s of cycling at 5 °C and 55 °C) is a recognized method for studying the effects of long-term denture use^25–28^. To validate the long-term clinical performance of newly introduced 3D-printed denture bases, further research is needed to evaluate the effects of conventional polymerization, milling, and 3D-printed resin materials in simulated oral environments. Therefore, this study aimed to assess the impact of thermal cycling on the surface roughness, hydrophilicity, and color stability of milled and 3D-printed denture base resin materials using conventional polymeric resins as a control. The study also investigated the color difference among the three materials in different staining media (distilled water, artificial saliva, Coca-Cola, and green tea). The hypotheses of this study are: (1) Thermal cycling has the same effect on surface roughness, hydrophilicity, and color stability across conventional, milled, and 3D-printed denture base resins. (2) There is no difference in the color difference values of the three groups after 10,000 thermal cycles and 7 or 30 days of immersion in different staining media.

## Materials and methods

### Data construction and sample fabrication

Rectangular samples (15 mm×15 mm×3 mm) were designed using UG 3D build software (Unigraphics NX, Siemens PLM Software, Plano, TX, USA) according to the ISO-20795-1: 2013 standard^29^. Sample sizes were calculated using G Power software (G Power 3.1.9.7, University of Düsseldorf, Düsseldorf, Germany) with an effect size of 0.25, statistical power (1-β) of 0.8, and a significance level of 0.05. For the roughness and hydrophilicity studies, the required sample size per group was calculated to be 12, which was increased to 15 per group in this study. For the color stability study in different staining media, the required sample size per group was calculated to be 9.38, with 10 samples per group used in the study. A total of 70 test samples of denture base resins were prepared using conventional thermal polymerization (conventional), CAD/CAM milling (milled), and 3D printing (3D-printed) methods. Detailed information on the three base resins is provided in Table 1.

**Table 1:** Three types of resin base materials used in this study.

Material	Fabrication technique	Composition	Production information
Denture base polymer	Conventional; heat-polymerized	Powder: polymethyl methacrylate, pigments Liquid: methyl methacrylate	Size: 1KG/pack Lot: 230,603 Manufacturer: Shanghai Pigeon Dental MFG Co. Ltd
Denture base resin discs for subtractive manufacturing	Milled	Polymethyl methacrylate, Methyl methacrylate, Ethylene glycol dimethacrylate, Pigments	Size: 25 mm thick,98 mm diameter disc Lot: 200,818,010 Manufacturer: Shandong Huge Dental Material Corporation
Fluid resins for additive manufacturing denture bases	3D-printed	Di-2-methylpropanoic acid acyloxyethyl-2,2,4-trimethylhexane di-carbamate, 1,6-Hexanediyl bis(2-methylacrylate), Diacrylic acid, diester with ,3’-(isopropylidene)bis(p-phenyleneoxy)di(propane-1,2-diol), Strontium glass powder, Barium glass powder, Silicon powder, The others(Camphorquinone, 4-Methoxyphenol, Ferric oxide)	Size: 1 kg/barrel Lot: YT2-231122 Manufacturer: Sino-Dentex Co. Ltd

Fabrication of Conventional Denture Base Resin Samples: First, an aluminum alloy sample was created by importing the design data into a 6-axis, 5-connected milling machine (308B, Willemin-Macodel SA, Switzerland) and programming the processing steps. This alloy sample was then used to create a silicone rubber negative mold, which was filled with molten wax to produce the final wax sample. The wax samples were processed using conventional techniques, including flasking, investing, wax removal, and resin material filling, to obtain the base resin samples. After removing any burrs, defect-free samples were produced (conventional group). Following the methods of previous researchers^22^, the base resin samples from the conventional group were polished under running water using 1000 mesh, 2000 mesh, and 5000 mesh water-abrasive sandpaper (Eagle, Suzhou Budweiser Polishing Materials Co., Ltd, Suzhou, China). Each grit was applied at a constant speed for 10 s. Only the upper surface of the samples was polished (simulating the polished surface), while the lower surface was left unprocessed (simulating the tissue surface). After polishing, the samples were ultrasonically cleaned with distilled water for 5 min and dried with sterile gauze. Finally, the consistency of the samples’ dimensions was verified using digital calipers, ensuring dimensional differences were ≤ 0.2 mm.

Fabrication of Milled Denture Base Samples: The design data were imported into a 6-axis, 5-connected milling machine (the same as used for the conventional group). After editing the processing program, a PMMA disc (16 mm thick, 98.5 mm in diameter) (7#A3-PINK, Shandong Huge Dental Material Corporation, Shandong, China) was processed to produce the samples (milled group). The processing parameters were as follows: ball-end tools were selected with sizes R2.0L15mm (T1), R1.0L12mm (T5), and R0.5L8mm (T7), with a new tool used for each group of PMMA resin disc samples. The tool and processing strategy were as follows: T1 for rough processing, S = 18,000 r/min, F = 2,000 mm/min; T5 for optimized semi-finish processing, S = 18,000 r/min, F = 1,000 mm/min; T7 for fine processing, S = 18,000 r/min, F = 800 mm/min; T5 for final cut-off support, S = 18,000 r/min, F = 1,000 mm/min. The disc fixture was selected with resin disc material specifications: thickness = 35 mm, diameter = 98.5 mm. The sample’s upper and lower surfaces were aligned perpendicularly to the disc plane, and the processing mode was set to automatic. After processing, a tungsten carbide acrylic drill was used to mill the denture base sample from the disc. The milled group was polished in the same manner as the conventional group.

**Fig. 1:**
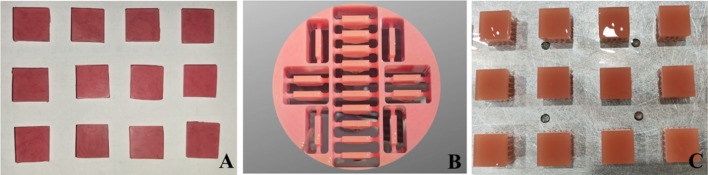
Partially processed base resin samples. (**A**) Conventional. (**B**) Milled. (**C**) 3D-Printed.

Fabrication of 3D-Printed Base Resin Samples: The design data were imported into a 3D printer (IBEE300, UNIZ Technology LLC, Beijing, China), and the matched printing material was used to fabricate the samples (3D-printed group). According to the manufacturer’s guidelines, the printing parameters were set as follows: LED light source wavelength of 405 nm and print layer thickness of 50 μm. After printing, the excess resin was cleaned with a 90% isopropyl alcohol solution, and the support structure was removed using a tungsten carbide acrylic drill and sandpaper. The samples were then post-cured for 5 min in a light-curing oven (UV OVEN, Prismlab China Co., Ltd, Shanghai, China) (405 nm) following the manufacturer’s instructions. Finally, the samples were polished in the same manner as those in the conventional and milled groups. The completed samples from the three groups are shown in Fig. 1.

### Surface roughness testing

Before thermal cycling, 15 samples were randomly selected from each of the three groups (from the total of 70 samples per group) for surface roughness measurement. Following a previous research^30^ method, four areas approximately 500 × 700 μm² in size were randomly selected on the polished surface of each sample before thermal cycling treatment. The surface roughness of each sample was examined using a morphological contour microscopic system (VK-X150 K, Keyence, Osaka, Japan) to detect the surface roughness index. Four non-adjacent typical areas with a size of 500 × 700 μm² were randomly selected for image scanning on each sample. The acquired raw 3D images were then processed through datum correction (surface undulation elimination), automatic noise elimination, and filter processing to obtain a magnified 400-fold 3D morphology image of the surface. Finally, the Sa value (which represents the absolute average difference in height of the points relative to the mean surface) was calculated by computer, representing the surface roughness of each group of samples.

One sample was randomly selected from each of the three groups. The surface of each sample was ultrasonically cleaned for 10 min and dried with sterile gauze, then evacuated and plated. The surface morphology of the samples before and after 10,000 thermal cycles was observed using a scanning electron microscope (SEM) (Tabletop Microscopes TM4000Plus, Hitachi High-Tech Co., Ltd, Tokyo, Japan) at 500x magnification and 5 kV voltage.

### Hydrophilicity analysis

Following a previous study^31^, the contact angle of the resin samples (*n* = 15) was measured using the sessile drop method (SDM) to assess contact angle. Before thermal cycling, the samples were placed on the stage of a contact angle meter (SCI3000F, Beijing Global Hengda Technology Co., Ltd, Beijing, China). A 1 ml drop of distilled water was placed onto the surface of each sample using a special syringe, and images were captured using the contact angle software. The images were then analyzed with ImageJ software (National Institutes of Health, Bethesda, USA) to measure the contact angle. Three drops were randomly placed on each sample, with each measurement taken once, and the mean value recorded. A larger contact angle indicated less hydrophilicity and more hydrophobicity.

Next, the three groups were placed in a high and low temperature exchanger (TC-501 F III, Suzhou Will Experimental Supplies Co., Ltd, Suzhou, China), with each group fully immersed in a bath of distilled water at 5℃ and 55℃ for 30 s each, and an automatic transfer time of 10 s. The same method was then used to measure the surface roughness and contact angle of each sample after 10,000 thermal cycles.

### Color stability analysis

Referring to a previous color evaluation method^19^, the accuracy of the colors was determined using the color space reference scale (1976 CIE Lab, International Commission on Illumination, Copyright© 2020 CIE). In this scale, L* represents luminance, with values ranging from 0 (black) to 100 (white); a* represents color saturation on the red-green axis, with -a* indicating green and + a* indicating red; b* represents color saturation on the blue-yellow axis, with -b* indicating blue and + b* indicating yellow. This scale represents the visible color spectrum as perceived by the human eye. The color of each group of samples was measured using a spectrophotometer (CM-700d, Konica Minolta, Tokyo, Japan) according to the manufacturer’s instructions. To ensure accurate color measurement, the spectrophotometer was calibrated with a white calibration plate (CMA145, Konica Minolta, Tokyo, Japan) before each use. The L, a, and b values were measured at a viewing angle of 10° under the illumination of a D65 light source (color temperature 6500 K, the international standard for daylight). Data for the L, a, and b values were collected using spectrophotometric measurement software (SpectraMagic Nx, Konica Minolta, Tokyo, Japan). The color values before thermal cycling were used as the reference, recorded as L_0_, a_0_, and b_0_. Each sample was measured three times, and the average value was recorded.

Next, three groups of samples (*n* = 40) were placed in the thermal cycling device (as above) for 5,000 and 10,000 cycles at a room temperature of 23 ± 2℃ (with cycling conditions as described previously). After thermal cycling, the color values of each sample were recorded separately (after 5,000 cycles as L_1_, a_1_, b_1_, and after 10,000 cycles as L_2_, a_2_, b_2_). The color measurements for each group were conducted as described earlier.

After 10,000 thermal cycles, the samples from each of the three groups were randomly divided into four subgroups (*n* = 10 per subgroup). These were fully immersed in an electric thermostat (303-0, Shaoxing Super Instrument Co., Ltd., Zhejiang, China) containing one of four staining media after being stored in distilled water at 37 ± 1 °C for 24 h. The media used were: distilled water (150427176 K; Nandai Industrial Co., Ltd., Zhejiang, China) (control group), tested according to GB/T6682-2008 standards, ‘Specification and Test Methods of Water for Analytical Laboratories’; artificial saliva (A7990, Beijing Solarbio Science & Technology Company, Beijing, China), composed of deionized water, NaCl, KCl, Na_2_SO_4_, NH_4_Cl, CaCl_2_, H2O, NaH_2_PO_4_, CN_2_H_4_O, and NaF; green tea (SC11441152200015; Rizhao Yuqing Tea Industry Co., Ltd, Rizhao, China), prepared by brewing 2 g of green tea in 300 ml of 100 °C hot water, then cooling to room temperature; and Coca-Cola (SC10650011210628, Coca-Cola Beverage Co., Ltd., Beijing, China), containing sugar, carbonated water (carbon dioxide and water), caramel, phosphoric acid, and caffeine. The samples were kept in a constant temperature water bath at 37 ± 1 °C to simulate the intraoral environment. As per previous studies^19^, the staining medium was changed every 24 h to prevent contamination. The color of each sample was measured on days 7 and 30 (recorded as L_3_, a_3_, b_3_, and L_4_, a_4_, b_4_, respectively). Before each measurement, the samples were ultrasonically cleaned with distilled water for 5 min and dried with sterile gauze. The color values were measured as previously described.

Finally, the color difference (∆E) values were calculated for each sample after 5,000 and 10,000 thermal cycles, and after 10,000 thermal cycles followed by immersion in the four different staining media for 7 and 30 days, respectively. The ∆E value was calculated using the formula :

$$\Delta {\text{E}} = \sqrt {\Delta {\text{L}}^{2} + \Delta {\text{a}}^{2} + \Delta {\text{b}}^{2} }$$

where ∆L = L*-L_0,_ ∆a = a*-a_0,_ ∆b = b*-b_0_. According to the ISO/TR 28,642: 2016 specification, a ∆E ≤ 1.2 indicates no significant color change, ∆E values between 1.2 and 2.7 are considered within clinically acceptable threshold, and ∆E > 2.7 is considered clinically unacceptable.

### Statistical analysis

Data were statistically analyzed using SPSS 27.0 software (SPSS 27.0, IBM SPSS Inc., Chicago, IL, USA). Normality was assessed using the Shapiro-Wilk test, and homogeneity of variances was checked using the Levene test. One-way ANOVA and Tukey’s test or the Kruskal-Wallis H test were employed to evaluate the surface roughness, hydrophilicity, and color stability of the three groups after different numbers of thermal cycles. Pearson or Spearman correlation analyses were conducted to examine the relationships between surface roughness and contact angles for the three groups before and after thermal cycling. Finally, a multifactor ANOVA was performed using fabrication method, staining medium, and immersion time as independent factors to analyze the color stability of the three groups. The significance level was set at α = 0.05.

## Results

### Surface morphology and roughness

**Fig. 2:**
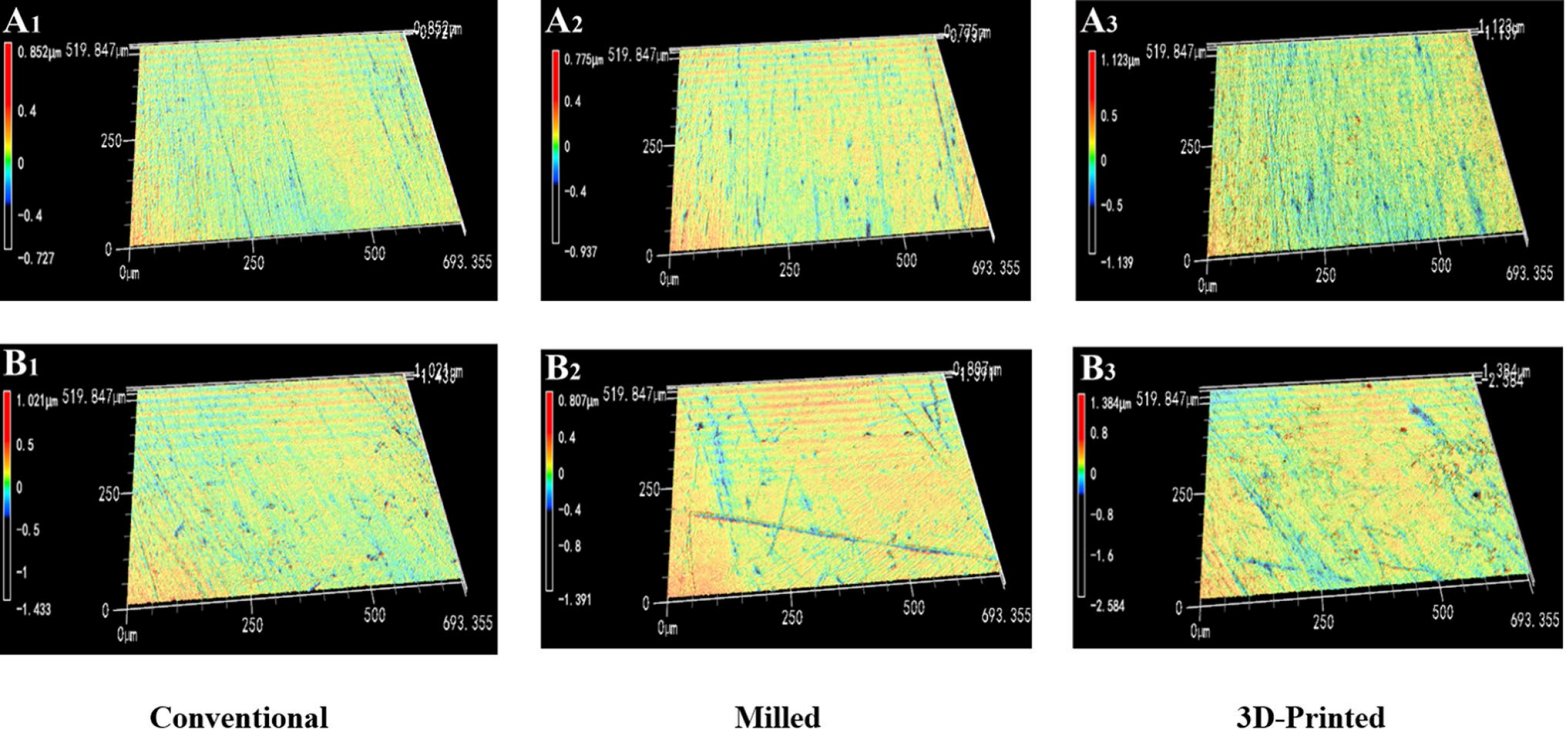
Typical morphology of samples before and after 10,000 thermal cycles in three groups. Before thermal cycling: (**A**_1_, **A**_2_, **A**_3_). After thermal cycling: (**B**_1_, **B**_2_, **B**_3_).

**Fig. 3:**
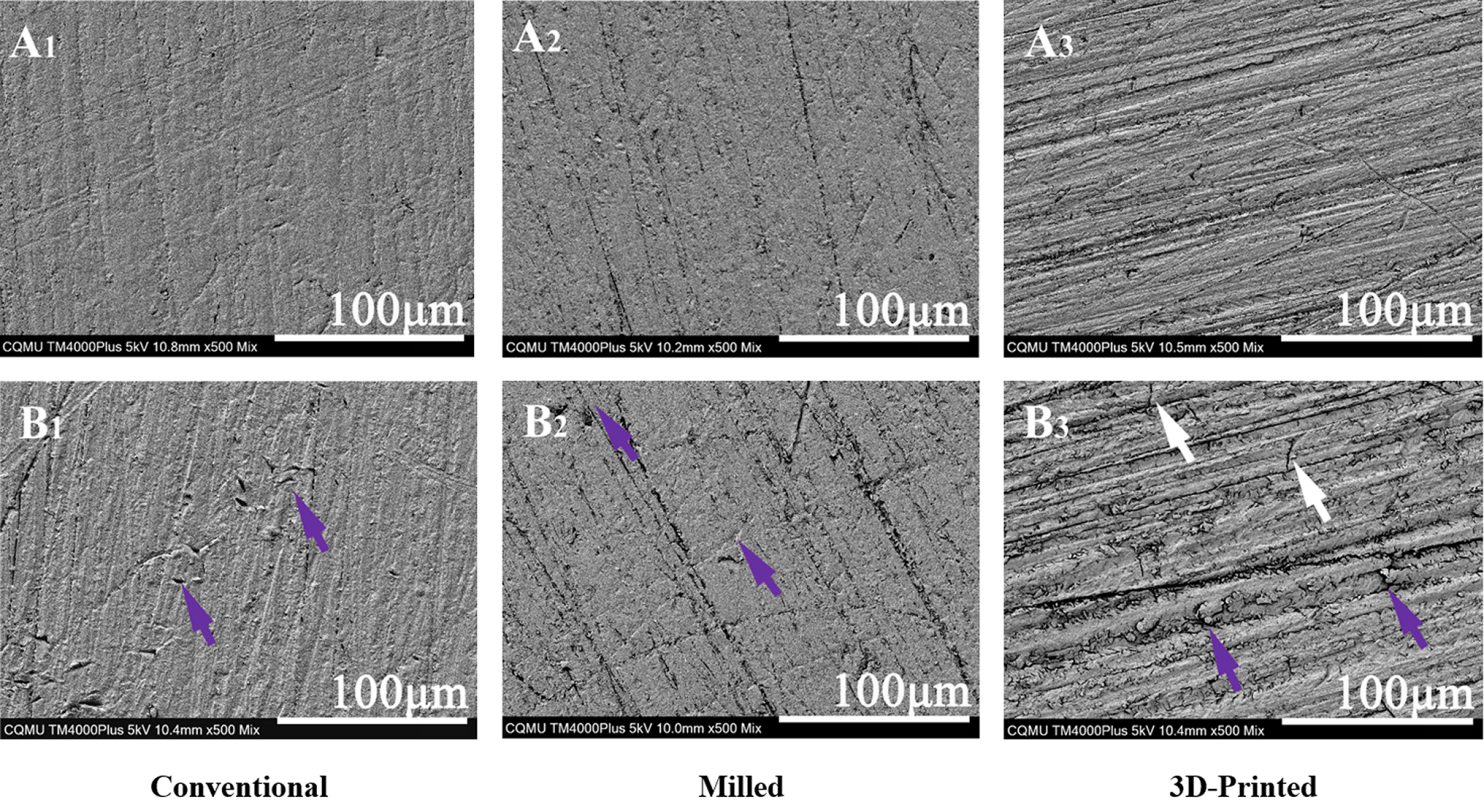
SEM images at 500× magnification of typical samples before and after 10,000 thermal cycles in three groups. Before thermal cycling: (**A**_1_, **A**_2_, **A**_3_. After thermal cycling: **B**_1_, **B**_2_, **B**_3_. Significant variations after thermal cycling are shown by arrows, purple arrows indicate defects and white arrows show cracks.

Figure 2 presents the typical three-dimensional surface morphology of the three groups. Before thermal cycling, the surface morphology of the conventional (Fig. 2, A1) and milled (Fig. 2, A2) groups appeared similar, with overall flatness interspersed with scratches of varying directions and lengths. The differences between the protrusions and depressions within the scratches were minimal. In contrast, the 3D-printed group exhibited a more uneven surface, characterized by more pronounced peaks and valleys, and greater height differences (Fig. 2, A3). After 10,000 thermal cycles, the overall morphology of the conventional (Fig. 2, B1) and milled (Fig. 2, B2) groups remained relatively uniform, though the scratches became more pronounced. The 3D-printed group, however, displayed a more irregular surface with the emergence of small protrusions and pit defects in certain areas (Fig. 2, B3). In the scanning electron microscope images, the surfaces of all three groups initially showed a scratch-like morphology, with scratches differing in shape and direction but generally appearing regular (Fig. 3, A1, A2, A3). After thermal cycling, the scratches on the conventional (Fig. 3, B1) and milled (Fig. 3, B2) groups became more prominent, fragmented, or irregular, with small defects or burrs visible in some areas. In contrast, the 3D-printed group displayed a clear fold-like morphology, with defects or cracks visible along the folds (Fig. 3, B_3_).

Table 2 presents the Sa values for the three groups before and after 10,000 thermal cycles. The 3D-printed group exhibited the highest Sa values both before and after thermal cycling compared to the conventional and milled groups. A significant difference in surface roughness was observed among the three groups before thermal cycling (*P* < 0.001), while no statistically significant difference was found between the conventional and milled groups after thermal cycling (*P* > 0.05). Following thermal cycling, Sa values increased in all three groups to varying degrees, with each group showing a statistically significant difference between the before and after measurements (*P* < 0.001).

**Table 2:** Surface roughness (Sa) values of samples before and after 10,000 thermal cycles (*n* = 15, µm).

Group	Conventional	Milled	3D-Printed	F	*P*
Before	0.07 ± 0.01^Cb^	0.10 ± 0.01^Bb^	0.21 ± 0.03^Ab^	379.40	< 0.001
After	0.13 ± 0.02^Ba^	0.14 ± 0.02^Ba^	0.48 ± 0.08^Aa^	406.62	< 0.001
F	242.40	49.95	199.06		
*P*	< 0.001	< 0.001	< 0.001		

Data following a normal distribution are expressed as “mean ± standard deviation.” Different superscript capital letters indicate significant differences among the three groups (*P* < 0.05), while different superscript lowercase letters indicate significant differences within the same group of samples (*P* < 0.05).

### Hydrophilicity

Table 3 shows the contact angles of the three groups before and after 10,000 thermal cycles. The results indicate that the contact angle in the 3D-printed group was significantly larger than in the conventional or milled groups both before and after thermal cycling (*P* < 0.05). However, the difference between the conventional and milled groups was not statistically significant (*P* > 0.05).

**Table 3:** Contact angle of samples before and after 10,000 thermal cycles in three groups (*n* = 15, degrees).

Group	Conventional	Milled	3D-Printed	F	*P*
Before	63.33 ± 0.67^Bb^	64.09 ± 0.80^Bb^	68.08 ± 1.12^Ab^	124.98	< 0.001
After	73.34 ± 0.64^Ba^	74.06 ± 0.84^Ba^	77.66 ± 1.16^Aa^	97.57	< 0.001
F	1748.06	1103.41	529.19		
*P*	< 0.001	< 0.001	< 0.001		

Data following a normal distribution are expressed as “mean ± standard deviation.” Different superscript capital letters indicate significant differences among the three groups (*P* < 0.05), while different superscript lowercase letters indicate significant differences within the same group of samples (*P* < 0.05).

Correlation analysis between Sa and contact angle (Table 4) revealed a statistically significant correlation between these two variables before and after thermal cycling for all three groups (*P* < 0.05). The correlation coefficients were positive, indicating that higher surface roughness was associated with larger contact angles, implying lower hydrophilicity.

**Table 4:** Correlation coefficients between surface roughness and contact angle for three groups of samples before and after 10,000 thermal cycles.

Group		Conventional	Milled	3D-Printed	Total
Before	r	0.61	0.56	0.62	0.89
	*P*	0.02	0.03	0.01	< 0.001
After	r	0.62	0.64	0.58	0.87
	*P*	0.01	0.01	0.02	< 0.001

### Color stability

#### Color difference values (ΔE ) after 5000 and 10,000 thermal cycles

The ∆E values for the three groups after 5,000 and 10,000 thermal cycles are presented in Table 5. The mean ∆E values after different numbers of cycles followed this order: 3D-printed group > conventional group > milled group. After 10,000 thermal cycles, the ∆E values in the 3D-printed group were significantly higher than those in the conventional or milled groups (*P* < 0.05), while no statistically significant difference was observed between the conventional and milled groups (*P* > 0.05). Additionally, the ∆E values increased significantly in all three groups after 5,000 and 10,000 thermal cycles (*P* < 0.05). In the milled group, the ∆E values after 5,000 and 10,000 cycles were below 1.2, indicating no significant color difference. In both the conventional and 3D-printed groups, the ∆E values after 5,000 and 10,000 cycles were below 2.7, indicating that the color differences were within the clinically acceptable threshold.

**Table 5:** Mean ∆E values for three groups of samples after 5,000 and 10,000 thermal cycles (*n* = 40).

Cycles	Conventional	Milled	3D-printed	F	*P*
5000	1.10 ± 0.39^Bb^	0.59 ± 0.30^Cb^	1.51 ± 0.42^Ab^	61.25	< 0.001
10,000	1.41 ± 0.66^Ba^	1.12 ± 0.47^Ba^	2.01 ± 0.78^Aa^	18.32	< 0.001
H	4.44	27.41	8.67		
*P*	0.035	< 0.001	0.003		

Data following a normal distribution are expressed as “mean ± standard deviation.” Different superscript capital letters indicate significant differences among the three groups after 5,000 or 10,000 thermal cycles (*P* < 0.05), while different superscript lowercase letters indicate significant differences within the same group of samples after 5,000 or 10,000 thermal cycles (*P* < 0.05).

#### Color difference values (ΔE) in different staining medium after 10,000 thermal cycles

**Fig. 4:**
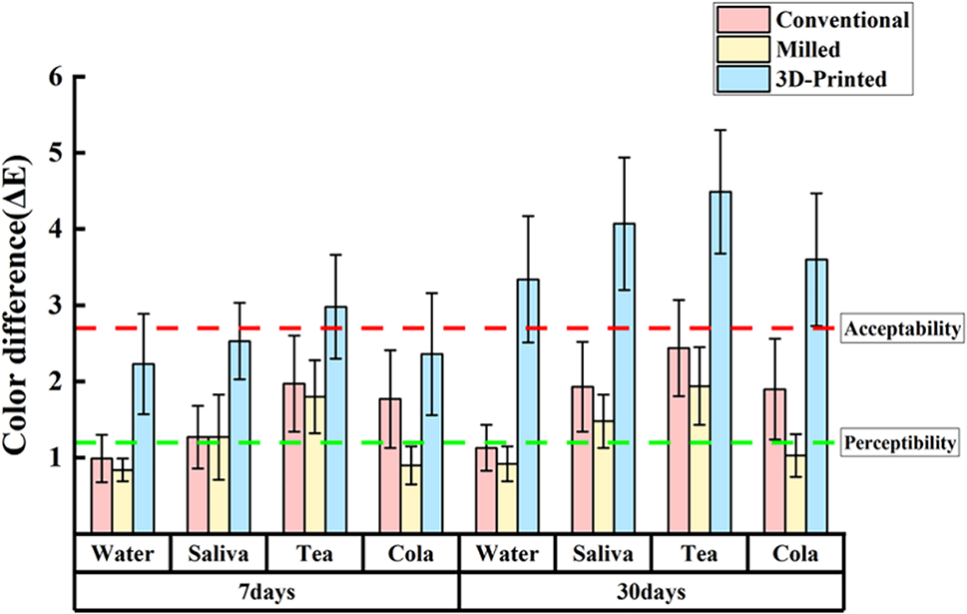
Mean ∆E ± standard deviation values for three groups of samples immersed in different staining medium on days 7 and 30. Water: distilled water. Saliva: artificial saliva. Tea: green tea. Cola: Coca-Cola.

Figure 4 shows the mean ∆E values for the three groups immersed in different staining media for 7 and 30 days after 10,000 thermal cycles. Among the four staining media at day 7 or day 30, green tea showed the highest mean ∆E value, followed by artificial saliva and Coca-Cola, with distilled water having the lowest ∆E value. In additional, the ∆E values for the 3D-printed group, both at 7 and 30 days, were significantly higher than those for the conventional and milled groups. The mean ∆E values for all three groups were significantly higher after 30 days of immersion compared to 7 days. At 30 days, the mean ∆E values for the 3D-printed group (∆E ≥ 3.34) exceeded the clinically acceptable threshold, whereas the mean ∆E values for the conventional and milled groups remained within the clinically acceptable threshold (∆E < 2.7).

**Fig. 5:**
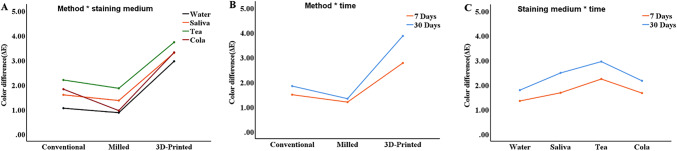
Interaction plot of color difference values (ΔE) by fabrication method, staining medium, and immersion time. (**A**) Interaction between fabrication method and staining medium. (**B**) Interaction between fabrication method and immersion time. (**C**) Interaction between staining medium and immersion time. Water: distilled water. Saliva: artificial saliva. Tea: green tea. Cola: Coca-Cola

Figure 5 illustrates the interaction effects of method, staining medium, and immersion time on mean ∆E values. In the interaction between method and staining medium, the 3D-printed group had the highest mean ∆E value across all four staining media, followed by the conventional group, with the milled group having the lowest (Fig. 5A). In the interaction between fabrication method and immersion time, the 3D-printed group again had the highest mean ∆E value at both day 7 and day 30, followed by the conventional group, with the milled group showing the lowest values (Fig. 5B). The interaction between staining medium and immersion time revealed that green tea had the highest mean ∆E value at both day 7 and day 30, followed by artificial saliva and Coca-Cola, while distilled water had the lowest (Fig. 5C).

## Discussion

In this study, denture resin samples were fabricated using conventional, milling, and 3D printing methods. The surface roughness, hydrophilicity, and color stability of the three groups were assessed after thermal cycling, which included factors such as fabrication method, staining medium, and immersion time at 5 °C and 55 °C to simulate the oral environment. The results indicated that the 3D-printed group exhibited greater roughness, lower hydrophilicity, and reduced color stability compared to the conventional and milled groups, both before and after thermal cycling. However, there were no significant differences in surface roughness, hydrophilicity, and color stability between the conventional and milled groups (or the differences remained within the clinically acceptable threshold). Therefore, the hypotheses of this study can only be partially rejected, as the samples tested in the milled group were not inferior to those in the conventional group, suggesting that the tested CAD/CAM subtractive manufacturing resins performed comparably to conventional resins.

In general, the smaller the surface roughness, the smoother the surface. A smooth restorative surface enhances patient comfort, as the tip of the tongue can detect roughness changes as small as 0.30 μm^32^. In this study, the Sa values for the three groups before thermal cycling ranged from 0.07 to 0.21 μm, which are below the roughness level detectable by the tongue. After 10,000 thermal cycles, the Sa values increased across all three groups, ranging from 0.13 to 0.48 μm, with the 3D-printed group exceeding the tongue’s perceptible roughness threshold. This suggests that thermal cycling affects the surface roughness of all three groups, with a more pronounced effect on the 3D-printed group compared to the conventional and milled denture base groups. This increase in roughness could potentially lead to greater patient discomfort after clinical use in the 3D-printed group compared to the conventional or milled groups. In this study, the thermal cycling effects on the conventional and milled groups remained at or below the clinical threshold of 0.2 μm described previously^33^. The Sa values of the conventional and milled groups before and after thermal cycling were significantly lower than those of the 3D-printed group, and repeated thermal cycling did not raise their Sa values above 0.2 μm. These results are consistent with previous findings^22,27^, indicating that the 3D-printed denture base in this study is less resistant to surface degradation over long-term use compared to the conventional and milled denture base materials. This difference can be attributed to the material properties and fabrication processes. The conventional group used a pre-polymerized PMMA material, while the milled PMMA discs were fabricated under high pressure and high temperature, resulting in lower residual monomer content and a higher degree of polymerization^10^. In contrast, the 3D-printed denture base samples were formed through a layer-by-layer stacking process and polymerized post-printing, which may lead to weaker inter-layer bonding due to unreacted residual monomers. This can result in lower or incomplete polymerization, more voids, and spacing between layers, all contributing to a rougher surface^34^. The varying surface roughness among the three groups in this study may also be influenced by the different processing techniques used for gritting and polishing. Gritting increases the surface roughness of the samples, while polishing processes may lead to the reorientation of the surface polymer chains^14^.

The contact angle is a parameter used to measure the hydrophilicity of a liquid on a solid surface, with a larger contact angle indicating a less hydrophilic surface. In this study, the mean contact angle of the conventional polymerization group, both before and after thermal cycling, did not significantly differ from that of the milled group, but it was significantly lower than that of the 3D-printed group. The contact angle values for the 3D-printed group were very similar to those reported in a previous study (70.20-73.44°)^18^, suggesting that the hydrophilicity of the 3D-printed group, before and after thermal cycling, was inferior to that of the conventional and milled groups. The contact angles of all three groups significantly increased (indicating decreased hydrophilicity) after thermal cycling, implying that thermal cycling had a notable impact on the hydrophilicity of all groups. This change may be attributed to the inherent characteristics of the surface, including the chemical composition, surface morphology, membrane medium, and charge of the samples^14,18^. When combined with the surface roughness data from this study, the findings suggest that higher surface roughness corresponds with a higher contact angle and lower hydrophilicity, consistent with the results of previous studies^14,35^. Specifically, as surface roughness increases, the hydrophobicity of the denture base resin also increases, while hydrophilicity decreases. Increased surface roughness enhances the hydrophilicity of hydrophilic surfaces when the liquid fully diffuses, but conversely, it increases the hydrophobicity of hydrophobic surfaces by maintaining the droplet shape. Therefore, the observed decrease in hydrophilicity in this study could be attributed to the larger surface area created by the increased surface roughness due to thermal cycling, which enhances the interaction between water droplets and the functional groups of the acrylic resins, thereby increasing hydrophobicity. It is important to note that, unlike the conventional and milled groups, which were primarily composed of PMMA, the main component of the denture base materials in the 3D-printed group was not PMMA. Instead, they contained a significant amount of additives (e.g., polymerization initiators, accelerators, cross-linking agents, fillers, and colorants) (Table 1), leading to inconsistencies in hydrophilicity among the three groups. Previous researches^16,36^ have shown that significant differences in free surface energy and wettability of different denture base materials are primarily due to these varying additives.

Color difference (∆E) is a critical clinical indicator of denture base aging and degradation, and it is a common reason for patients to seek denture replacement. Artificial thermal cycling can simulate the thermal stresses that base resin materials endure when exposed to hot and cold beverages in the oral environment. To approximate clinical conditions more closely, this study first subjected each group to 5,000 and 10,000 thermal cycles, then immersed them in four different staining media for 7 and 30 days. The combined effects of thermal cycling, staining medium, and immersion time on the color stability of the denture bases were subsequently evaluated. In this study, ∆E values increased to varying degrees in all three groups after 5,000 and 10,000 thermal cycles. However, the ∆E value for the milled group remained below 1.2 after both 5,000 and 10,000 cycles, indicating no significant color difference. The ∆E values for the conventional group (∆E < 1.5) and the 3D-printed group after 5,000 and 10,000 cycles were below 2.7, meaning the color differences were within the clinically acceptable threshold. 10,000 thermal cycles are roughly equivalent to one year of clinical denture use^6,37^. This suggests that while thermal cycling over approximately one year does affect the color stability of all three groups to some extent, the impact is limited, and none of the denture bases exceeded the clinically acceptable threshold. However, the ∆E value for the 3D-printed group is approaching the clinically acceptable threshold (∆E = 2.7). This result indicates that with additional cycles, the 3D-printed group may not meet clinical requirements, suggesting that its color stability may be less reliable than that of the conventional or milled groups.

Further immersion of the three groups in distilled water, artificial saliva, green tea, and Coca-Cola for 7 and 30 days revealed that the mean ∆E values of all three groups significantly increased after 30 days compared to 7 days. Notably, the mean ∆E values for the 3D-printed group (∆E ≥ 3.34) greatly exceeded the clinically acceptable threshold after 30 days, while the mean ∆E values for the conventional and milled groups remained within the clinically acceptable threshold (∆E < 2.7) at both 7 and 30 days. The results regarding fabrication method and immersion time align with or are consistent with findings from previous studies^19,24^. These results suggest that color stability worsens with increased immersion time, and the potential negative impact on color stability is more pronounced in the 3D-printed group, which surpasses the clinically acceptable threshold. One study^18^ indicated that surface smoothness or roughness directly affects susceptibility to exogenous staining. In this study, the roughness of the 3D-printed group before and after 5,000 or 10,000 thermal cycles was significantly higher than that of the conventional or milled groups, supporting these findings. Additionally, 3D-printed resin typically requires low viscosity, necessitating reduced inorganic filler particles. A lower content of inorganic fillers reduces the wear resistance of resin materials, leading to surface deterioration during thermal cycling^28,38^. Furthermore, 3D-printed resins may experience filler particle settling during storage, resulting in a non-homogeneous layer of filler particles, which can cause improper polymerization and increased surface degradation during 3D printing^19,39^. Water absorption is another important factor affecting the color stability of resins^20^. Berli et al.^40^ demonstrated that water absorption in 3D-printed bases significantly increased after thermal cycling compared to conventional hot pressure casting or milled groups. Hanno KI et al.^26^ also found that 3D-printed resin exhibits higher water absorption due to increased residual monomer content and greater void and inter-layer spacing. The presentation of color is essentially the result of light reflection and absorption, and the optical properties of resin materials change with water absorption. A resin material with higher water absorption can also absorb more colored staining medium, leading to discoloration^27,41^. It can be speculated that the poorer color stability observed in the 3D-printed group in this study may be due to the absorption of more colored staining medium. However, it is important to note that there is no direct evidence from this study to suggest that the 3D-printed group had a higher residual monomer content than the conventional or milled groups. Therefore, further targeted studies are needed to validate this speculation.

Unlike many previous studies^12,19,20^, this study included green tea as a colored staining medium, commonly consumed by East Asian populations. The findings revealed that green tea resulted in the highest mean ∆E value among the staining media, followed by artificial saliva and Coca-Cola, with distilled water showing the lowest ∆E values. It is understandable that artificial saliva and Coca-Cola have higher coloring potential than distilled water. The stronger staining effect of green tea compared to artificial saliva or Coca-Cola is consistent with previous studies^42,43^. Although Coca-Cola is the most acidic solution and can cause degradation of the material surface, the higher staining effect of green tea may be attributed to its components. Tea contains a high concentration of flavonoids, which have been reported to contribute to discoloration^44^. Additionally, tea contains phenolic compounds, which are highly polar. Under certain conditions, these polar compounds can weaken the chemical bonds in acrylic resin, affecting the secondary polymer chain and increasing water absorption^45^. As discussed earlier, increased water absorption allows the material to absorb more of the colored staining medium, leading to a greater color difference. It is well known that when light interacts with an object, part of the light is absorbed, and the unabsorbed light is reflected. The wavelength of the reflected light determines the color observed. In this study, the components in green tea also absorb blue light, reflecting its complementary color, orange-yellow, which causes the material infused with green tea to exhibit a yellowish tint, thereby increasing its b-value. In contrast, Coca-Cola, which derives its color from added caramel, ranges from pale yellow to dark brown but lacks yellow pigment ^42,46,47^. Similarly, artificial saliva, despite being a solution composed of various chemicals, lacks yellow pigment. Therefore, both Coca-Cola and artificial saliva exhibit less staining than green tea.

Although the results of this study are significant, there are some limitations that should be acknowledged. First, the experimental samples were simple rectangular shapes rather than complete denture bases, which may not fully represent clinical conditions. Future studies could utilize resin models of intact bases to better assess the impact of thermal cycling on surface properties. Additionally, this remained an in vitro experiment. While efforts were made to simulate oral environmental conditions, the study could not fully replicate the complexities of actual patient factors like oral pH, enzymes, saliva flow and solution media types. Furthermore, the study did not account for materials’ resistance to microbial colonization in the oral environment, which is essential in long-term denture wear. And there is also a lack of patient-related consideration, such as the overall comfort or retention of the dentures, which are significant in clinical practice. Future research should aim to more accurately simulate the oral environment and design experimental protocols with greater precision to evaluate the combined effects of these factors on the surface properties and color stability of denture bases.

## Conclusions

The following conclusions can be drawn within this study:

1. The surface roughness of the 3D-printed group, both before and after 10,000 thermal cycles, was greater than that of the conventional or milled groups. The surface roughness increased in all three groups after thermal cycling.
2. The contact angle of the three groups, from largest to smallest, was as follows: 3D-printed group > milled group > conventional group, both before and after 10,000 thermal cycles. After thermal cycling, the contact angles of all three groups increased to varying degrees, indicating a decrease in hydrophilicity.
3. There was a clear positive correlation between surface roughness (Sa) and contact angle in all three groups, both before and after 10,000 thermal cycles. Specifically, larger surface roughness was associated with larger contact angles and a tendency toward lower hydrophilicity.
4. After 5,000 and 10,000 thermal cycles, the ∆E values of all three groups increased, with the 3D-printed group (∆E < 2.7) showing greater color change than the conventional or milled groups (∆E < 1.5). However, the color differences remained within clinically acceptable threshold.
5. After 10,000 thermal cycles, the ∆E values of the 3D-printed group were significantly higher than those of the conventional or milled groups after 7 or 30 days of immersion in the four different staining media. The mean ∆E values of all three groups were significantly higher after 30 days of immersion compared to 7 days. At 30 days, the mean ∆E values of the 3D-printed group (∆E ≥ 3.34) exceeded clinically acceptable threshold (∆E = 2.7), while the conventional and milled groups remained within the acceptable threshold at both 7 and 30 days.

In summary, thermal cycling or staining medium increased the surface roughness and decreased the hydrophilicity and color stability of conventional, milled, and 3D-printed denture base resin samples. However, the 3D-printed group exhibited a rougher surface, poorer hydrophilicity, and reduced color stability compared to the conventional or milled groups, indicating that further improvements are needed before clinical application.

## Electronic supplementary material

Below is the link to the electronic supplementary material.

Supplementary Material 1

## Data Availability

All data generated or analyzed in this study are included in this article and its supplementary information file. Any questions about the data in this study can be addressed to the first or corresponding author.

## References

[1] Srinivasan, M. et al. CAD-CAM complete denture resins: An evaluation of biocompatibility, mechanical properties, and surface characteristics. *J. Dent.* **114**, 103785. 10.1016/j.jdent.2021.103785 (2021). 34419480 10.1016/j.jdent.2021.103785

[2] Parvizi, A. et al. Comparison of the dimensional accuracy of injection-molded denture base materials to that of conventional pressure-pack acrylic resin. *J. Prosthodont.* **13**(2), 83–89. 10.1111/j.1532-849X.2004.04014.x (2004). 15210003 10.1111/j.1532-849X.2004.04014.x

[3] Revilla-León, M. & Özcan, M. Additive Manufacturing technologies used for Processing polymers: Current status and potential application in Prosthetic Dentistry. *J. Prosthodont.* **28**(2), 146–158. 10.1111/jopr.12801 (2019). 29682823 10.1111/jopr.12801

[4] Chen, J. et al. Shape optimization for additive manufacturing of removable partial dentures–a new paradigm for prosthetic CAD/CAM. *PLoS One*. **10**(7), e0132552. 10.1371/journal.pone.0132552 (2015). 26161878 10.1371/journal.pone.0132552 PMC4498620

[5] Hada, T. et al. Effect of printing direction on the accuracy of 3D-printed dentures using stereolithography technology. *Mater. (Basel)*. **13**(15), 3405. 10.3390/ma13153405 (2020). 10.3390/ma13153405 PMC7435373 32748815

[6] Gad, M. M. et al. Strength and Surface properties of a 3D-Printed denture base polymer. *J. Prosthodont.* **31**(5), 412–418. 10.1111/jopr.13413 (2022). 34347351 10.1111/jopr.13413

[7] Gad, M. M., Bağış, B. & Turgut, S. Effects of thermal cycling on surface roughness, hardness and flexural strength of polymethylmethacrylate and polyamide denture base resins. *J. Appl. Biomater. Funct. Mater.* **13**(3), e280–e286. 10.5301/jabfm.5000236 (2015). 26350350 10.5301/jabfm.5000236

[8] Silva Cde, S., Machado, A. L., Chaves Cde, A., Pavarina, A. C. & Vergani, C. E. Effect of thermal cycling on denture base and autopolymerizing reline resins. *J. Appl. Oral Sci.* **21**(3), 219–224. 10.1590/1679-775720130061 (2013). 23857648 10.1590/1679-775720130061 PMC3881901

[9] Morresi, A. L. et al. Thermal cycling for restorative materials: Does a standardized protocol exist in laboratory testing? A literature review. *J. Mech. Behav. Biomed. Mater.* **29**, 295–308. 10.1016/j.jmbbm (2014). 24135128 10.1016/j.jmbbm.2013.09.013

[10] Arslan, M., Murat, S., Alp, G. & Zaimoglu, A. Evaluation of flexural strength and surface properties of prepolymerized CAD/CAM PMMA-based polymers used for digital 3D complete dentures. *Int. J. Comput. Dent.* **21**(1), 31–40 (2018). 29610779

[11] Wei, X. et al. In vitro study of surface properties and microbial adhesion of various dental polymers fabricated by different manufacturing techniques after thermocycling. *Clin. Oral Investig*. **26**(12), 7287–7297. 10.1007/s00784-022-04689-2 (2022). 35976495 10.1007/s00784-022-04689-2

[12] Taşın, S., Ismatullaev, A. & Usumez, A. Comparison of surface roughness and color stainability of 3-dimensionally printed interim prosthodontic material with conventionally fabricated and CAD-CAM milled materials. *J. Prosthet. Dent.* **128**(5), 1094–1101. 10.1016/j.prosdent.2021.01.027 (2022). 33715836 10.1016/j.prosdent.2021.01.027

[13] Nam, N. E., Shin, S. H., Lim, J. H., Shim, J. S. & Kim, J. E. Effects of artificial tooth brushing and hydrothermal aging on the mechanical properties and color stability of dental 3D printed and CAD/CAM materials. *Mater. (Basel)*. **14**(20), 6207. 10.3390/ma14206207 (2021). 10.3390/ma14206207 PMC8540203 34683798

[14] de Foggi, C. C. et al. Effect of surface roughness on the hydrophobicity of a denture-base acrylic resin and Candida albicans colonization. *J. Investig Clin. Dent.* **7**(2), 141–148. 10.1111/jicd.12125 (2016). 25329611 10.1111/jicd.12125

[15] Ramiasa, M., Ralston, J., Fetzer, R. & Sedev, R. The influence of topography on dynamic wetting. *Adv. Colloid Interface Sci.* **206**, 275–293. 10.1016/j.cis.2013.04.005 (2014). 23726301 10.1016/j.cis.2013.04.005

[16] Steinmassl, O., Dumfahrt, H., Grunert, I. & Steinmassl, P. A. Influence of CAD/CAM fabrication on denture surface properties. *J. Oral Rehabil*. **45**(5), 406–413. 10.1111/joor.12621 (2018). 29473188 10.1111/joor.12621

[17] Al-Dwairi, Z. N., Tahboub, K. Y., Baba, N. Z., Goodacre, C. J. & Özcan, M. A comparison of the surface properties of CAD/CAM and conventional polymethylmethacrylate (PMMA). *J. Prosthodont.* **28**(4), 452–457. 10.1111/jopr.13033 (2019). 30730086 10.1111/jopr.13033

[18] Al-Dwairi, Z. N., Al Haj Ebrahim, A. A. & Baba, N. Z. A comparison of the surface and mechanical properties of 3D printable denture-base resin material and conventional polymethylmethacrylate (PMMA). *J. Prosthodont.* **32**(1), 40–48. 10.1111/jopr.13491 (2023). 35119168 10.1111/jopr.13491

[19] Gruber, S., Kamnoedboon, P., Özcan, M. & Srinivasan, M. CAD/CAM complete denture resins: An in vitro evaluation of Color Stability. *J. Prosthodont.* **30**(5), 430–439. 10.1111/jopr.13246 (2021). 32864812 10.1111/jopr.13246

[20] Alfouzan, A. F. et al. Color stability of 3D-printed denture resins: Effect of aging, mechanical brushing and immersion in staining medium. *J. Adv. Prosthodont.* **13**(3), 160–171. 10.4047/jap.2021.13.3.160 (2021). 34234926 10.4047/jap.2021.13.3.160 PMC8250187

[21] Çakmak, G. et al. Effect of simulated brushing and disinfection on the surface roughness and color stability of CAD-CAM denture base materials. *J. Mech. Behav. Biomed. Mater.* **134**, 105390. 10.1016/j.jmbbm.2022.105390 (2022). 35917636 10.1016/j.jmbbm.2022.105390

[22] Çakmak, G. et al. Surface roughness and color stability of 3D-Printed denture base materials after simulated brushing and thermocycling. *Mater. (Basel)*. **15**(18), 6441. 10.3390/ma15186441 (2022). 10.3390/ma15186441 PMC9503686 36143757

[23] Fouda, S. M. et al. Influence of denture brushing on the surface properties and color stability of CAD-CAM, thermoformed, and conventionally fabricated denture base resins. *J. Prosthodont.*10.1111/jopr.13801 (2023). Epub ahead of print. 37953735 10.1111/jopr.13801

[24] Takhtdar, M., Azizimoghadam, N., Kalantari, M. H. & Mohaghegh, M. Effect of denture cleansers on color stability and surface roughness of denture bases fabricated from three different techniques: Conventional heat-polymerizing, CAD/CAM additive, and CAD/CAM subtractive manufacturing. *Clin. Exp. Dent. Res.* **9**(5), 840–850. 10.1002/cre2.763 (2023). 37438935 10.1002/cre2.763 PMC10582232

[25] Tasin, S. & Ismatullaev, A. Comparative evaluation of effect of thermocycling on the mechanical properties of conventionally polymerized, CAD-CAM milled and 3D-printed materials. *J. Prosthet. Dent.* **127**, 173–179. 10.1016/j.prosdent.2021.09.020 (2022). 10.1016/j.prosdent.2021.09.020 34756771

[26] Hanno, K. I. & Abdul-Monem, M. M. Effect of denture cleansers on the physical and mechanical properties of CAD-CAM milled and 3D printed denture base materials: An in vitro study. *J. Prosthet. Dent.* **130**(5), 798e1–e8. 10.1016/j.prosdent.2023.08.009 (2023). 10.1016/j.prosdent.2023.08.009 37716896

[27] Abdul-Monem, M. M. & Hanno, K. I. Effect of thermocycling on surface topography and fracture toughness of milled and additively manufactured denture base materials: An in-vitro study. *BMC Oral Health*. **24**(1), 267. 10.1186/s12903-024-03991-7 (2024). 38395828 10.1186/s12903-024-03991-7 PMC10885363

[28] Çakmak, G. et al. Effect of coffee thermocycling on the surface roughness and stainability of denture base materials with different chemical compositions manufactured with additive and subtractive technologies. *J. Esthet Restor. Dent.* **36**(3), 453–459. 10.1111/jerd.13136 (2024). 37705502 10.1111/jerd.13136

[29] ISO-20795-1. Dentistry—denture base polymers. *Int. Organ. Stand.*, 6–7. (2013). https://www.iso.org/standard/62277.html

[30] Tong, X. L., Ma, C. Y., Yu, N., Zhou, H. Q. & Tan, F. B. Evaluation on surface characteristics, accuracy, and dimensional stability of tooth preparation dies fabricated by conventional gypsum and 3D-Printed Workflows. *Int. J. Prosthodont.* **0**, 1–32. 10.11607/ijp.8602 (2024). Epub ahead of print. 38408132 10.11607/ijp.8602

[31] Atalay, S., Çakmak, G., Fonseca, M., Schimmel, M. & Yilmaz, B. Effect of thermocycling on the surface properties of CAD-CAM denture base materials after different surface treatments. *J. Mech. Behav. Biomed. Mater.* **121**, 104646. 10.1016/j.jmbbm.2021.104646 (2021). 34166873 10.1016/j.jmbbm.2021.104646

[32] Jones, C. S., Billington, R. W. & Pearson, G. J. The in vivo perception of roughness of restorations. *Br. Dent. J.* **196**(1), 42–45. 10.1038/sj.bdj.4810881 (2004). 14966503 10.1038/sj.bdj.4810881

[33] Bollen, C. M., Lambrechts, P. & Quirynen, M. Comparison of surface roughness of oral hard materials to the threshold surface roughness for bacterial plaque retention: A review of the literature. *Dent. Mater.* **13**(4), 258–269. 10.1016/s0109-5641(97)80038-3 (1997). 11696906 10.1016/s0109-5641(97)80038-3

[34] El Samahy, M. M., Abdelhamid, A. M., El Shabrawy, S. M. & Hanno, K. I. Evaluation of physicomechanical properties of milled versus 3D-printed denture base resins: A comparative in vitro study. *J. Prosthet. Dent.* **129**(5), 797e1–e7. 10.1016/j.prosdent.2023.03.017 (2023). 10.1016/j.prosdent.2023.03.017 37121625

[35] Nishioka, M., Yamabe, Y., Hisatsune, K. & Fujii, H. Influence of polishing of denture base resin and metal surfaces on wettability with water and saliva. *Dent. Mater. J.* **25**(1), 161–165. 10.4012/dmj.25.161 (2006). 16706312 10.4012/dmj.25.161

[36] Sipahi, C., Anil, N. & Bayramli, E. The effect of acquired salivary pellicle on the surface free energy and wettability of different denture base materials. *J. Dent.* **29**(3), 197–204. 10.1016/s0300-5712(01)00011-2 (2001). 11306161 10.1016/s0300-5712(01)00011-2

[37] Gale, M. S. & Darvell, B. W. Thermal cycling procedures for laboratory testing of dental restorations. *J. Dent.* **27**(2), 89–99. 10.1016/s0300-5712(98)00037-2 (1999). 10071465 10.1016/s0300-5712(98)00037-2

[38] Diken Türksayar, A. A. & Baytur, S. Color stability, surface roughness and flexural strength of additively manufactured and milled interim restorative materials after aging. *Odontology* **111**(3), 680–686. 10.1007/s10266-022-00778-6 (2023). 36528659 10.1007/s10266-022-00778-6

[39] Vallittu, P. K., Ruyter, I. E. & Buykuilmaz, S. Effect of polymerization temperature and time on the residual monomer content of denture base polymers. *Eur. J. Oral Sci.* **106**(1), 588–593. 10.1046/j.0909-8836.1998.eos106109.x (1998). 9527360 10.1046/j.0909-8836.1998.eos106109.x

[40] Berli, C. et al. Comparing the mechanical properties of pressed, milled, and 3D-printed resins for occlusal devices. *J. Prosthet. Dent.* **124**(6), 780–786. 10.1016/j.prosdent.2019.10.024 (2020). 31955837 10.1016/j.prosdent.2019.10.024

[41] Gad, M. M., Al-Thobity, A. M., Fouda, S. M., Näpänkangas, R. & Raustia, A. Flexural and surface properties of PMMA denture base material modified with thymoquinone as an antifungal agent. *J. Prosthodont.* **29**(3), 243–250. 10.1111/jopr.12967 (2020). 30178899 10.1111/jopr.12967

[42] Ertaş, E., Güler, A. U., Yücel, A. C., Köprülü, H. & Güler, E. Color stability of resin composites after immersion in different drinks. *Dent. Mater. J.* **25**(2), 371–376 (2006). 16916243

[43] Shishehian, A. et al. Evaluating the color stability of 3D-printed resins against various solutions. *Eur. J. Transl Myol*. **33**(3), 11493. 10.4081/ejtm.2023.11493 (2023). 37767891 10.4081/ejtm.2023.11493 PMC10583149

[44] Imirzalioglu, P., Karacaer, O., Yilmaz, B., & Ozmen, I. Color stability of denture acrylic resins and a soft lining material against tea, coffee, and nicotine. *J. Prosthodont.* **19**(2), 118–124. 10.1111/j.1532-849X.2009.00535.x (2010). 20002978 10.1111/j.1532-849X.2009.00535.x

[45] Choure, R. B. et al. Effect of alcohol and tea on solubility of soft-liner and polymethyl methacrylate resin: An in vitro study. *J. Contemp. Dent. Pract.* **20**(1), 83–88 (2019). PMID: 31058618. 31058618

[46] Patel, S. B., Gordan, V. V., Barrett, A. A., & Shen, C. The effect of surface finishing and storage solutions on the color stability of resin-based composites. *J. Am. Dent. Assoc.* **135**(5), 587 – 94 (2004). 10.14219/jada.archive.2004.0246. PMID: 15202750. 10.14219/jada.archive.2004.0246 15202750

[47] Bagheri, R., Burrow, M.F., Tyas, M. Influence of food-simulating solutions and surface finish on susceptibility to staining of aesthetic restorative materials. *J. Dent.* **33**(5), 389–8. 10.1016/j.jdent (2005). 10.1016/j.jdent.2004.10.018 15833394

